# Accelerated long-term forgetting in aging and intra-sleep awakenings

**DOI:** 10.3389/fpsyg.2013.00750

**Published:** 2013-10-16

**Authors:** Alison Mary, Svenia Schreiner, Philippe Peigneux

**Affiliations:** ^1^UR2NF - Neuropsychology and Functional Neuroimaging Research Unit at CRCN - Center for Research in Cognition and Neurosciences, Université Libre de BruxellesBrussels, Belgium; ^2^UNI - ULB Neurosciences Institute, Université Libre de BruxellesBrussels, Belgium

**Keywords:** associative learning, accelerated long-term forgetting, declarative memory consolidation, aging, sleep

## Abstract

The architecture of sleep and the functional neuroanatomical networks subtending memory consolidation processes are both modified with aging, possibly leading to accelerated forgetting in long-term memory. We investigated associative learning and declarative memory consolidation processes in 16 young (18–30 years) and 16 older (65–75 years) healthy adults. Performance was tested using a cued recall procedure at the end of learning (immediate recall), and 30 min and 7 days later. A delayed recognition test was also administered on day 7. Daily sleep diaries were completed during the entire experiment. Results revealed a similar percentage of correct responses at immediate and 30-min recall in young and older participants. However, recall was significantly decreased 7 days later, with an increased forgetting in older participants. Additionally, intra-sleep awakenings were more frequent in older participants than young adults during the seven nights, and were negatively correlated with delayed recall performance on day 7 in the older group. Altogether, our results suggest a decline in verbal declarative memory consolidation processes with aging, eventually leading to accelerated long-term forgetting indicating that increased sleep fragmentation due to more frequent intra-sleep awakenings in older participants contribute to the reported age-related decline in long-term memory retrieval. Our results highlight the sensitivity of long-term forgetting measures to evidence consolidation deficits in healthy aging.

## INTRODUCTION

Memory decline is one of the most frequent complaints in the elderly (Craik and Rose, 2012). Accordingly, an age-related decline in memory performance has been mostly reported in the episodic memory domain (Nilsson, 2003). Episodic memory stores detailed information about personal events and allows recollection or conscious retrieval of these events within their spatial and temporal contexts (Tulving, 1972,^2002^). The formation of episodic memories is thought to rely on strategic and associative processes subtended by the frontal and medial temporal lobes (MTLs), respectively (Shing et al., 2010). Associative learning deficits have been often reported in aging (Chalfonte and Johnson, 1996; Naveh-Benjamin et al., 2003; Lowndes et al., 2008), leading to the associative deficit hypothesis (ADH) which explains age-related declines in episodic memory (Naveh-Benjamin, 2000). Within the ADH framework, memory decline results from a deficit in binding together the different features of a memory episode into a coherent representation. However, associative deficits might be related to the difficulties experienced by older adults to use efficient strategic processes at both encoding and retrieval (Dunlosky et al., 2005; Naveh-Benjamin et al., 2007). Therefore, a decline in the efficiency of declarative memory would be a normal component of the healthy aging processes, a decline that might be accelerated further in pathological aging. In this respect, accelerated long-term forgetting (ALF) was initially described in patients with epilepsy who exhibit normal performance in long-term memory and exaggerated forgetting when retested weeks later (Blake et al., 2000; Butler and Zeman, 2008). The ALF phenomenon suggests that the impairment in memory results from a consolidation deficit. Indeed, some patients with memory complaints can learn and accurately recall information for a limited period of time up to a few hours, but then exhibit poor recall after days to weeks following learning (Butler and Zeman, 2008). In Alzheimer’s disease, disturbances in memory consolidation processes can be observed after short delays, with an aggravated loss of information the first few minutes up to 1 h following the acquisition phase (Carlesimo et al., 1993; Larrabee et al., 1993; Christensen et al., 1998; Reed et al., 1998; Kramer et al., 2004). At variance, otherwise healthy memory clinic patients with memory complaints syndrome can achieve a normal performance at immediate and 30-min recall in verbal and visual memory tasks, but exhibit ALF after 6 weeks (Manes et al., 2008). These patients exhibit a similar long-term forgetting rate than patients presenting an amnestic mild cognitive impairment (aMCI), suggesting the possible existence of earlier stages in the processes evolving toward pathological aging. These results also highlight the crucial importance to test ALF to evidence early consolidation deficits, as may be seen in normal aging.

Accelerated forgetting is consistent with the dynamic views of memory consolidation processes which propose that novel and fragile memory traces are gradually transformed into a more stable representation that is more resistant to interference (McGaugh, 2000; Dudai, 2004; Frankland and Bontempi, 2005). This gradual process of memory consolidation is subtended by the progressive reorganization of memory-related neuroanatomical patterns during post-training sleep (Peigneux et al., 2003,^2004^; Huber et al., 2004; Walker and Stickgold, 2006; Rauchs et al., 2008; Diekelmann and Born, 2010) and wakefulness (Peigneux et al., 2006; Albert et al., 2009; Lewis et al., 2009; Tambini et al., 2010; Wang et al., 2012). The system consolidation hypothesis proposes that sleep-dependent consolidation of declarative memories is subtended by the reactivation of the novel memory traces initially stored in the hippocampus, followed by an hippocampal–neocortical dialog promoting the reorganization and integration of the new memories in neocortical structures for long-term memory storage (Buzsaki, 1996; Dudai, 2004), a process that can last for weeks to months to be achieved (Dudai, 2004). Considering that aging is associated with structural and functional changes in the MTL and prefrontal cortex (see Leal and Yassa, 2013; Maillet and Rajah, 2013, for reviews), these changes might contribute to memory consolidation deficits, especially for hippocampus-dependent declarative memories.

Substantial changes in sleep quality, quantity, and architecture occur in aging (Ohayon et al., 2004; Espiritu, 2008). Age-related changes in sleep architecture are characterized by decreased time spent in slow wave sleep (SWS) and rapid eye movement (REM) sleep, paralleled by increased time in light sleep (i.e., non-REM stage 1 and stage 2 sleep; Ohayon et al., 2004, for a meta-analysis). Additionally, total sleep time decreases whereas intra-sleep awakenings or wake after sleep onset (WASO) increases, thereby contributing to an age-related decline in sleep efficiency (Ohayon et al., 2004).

Therefore, both age-related changes in sleep patterns and in consolidation-related functional neuroanatomical networks may compromise long-term memory consolidation processes, and contribute to an accelerated long-term memory forgetting in aging (Mander et al., 2013). Accordingly, several studies have reported a lack of sleep-dependent consolidation for procedural learning in older healthy participants as compared to young adults (Spencer et al., 2007; Brown et al., 2009; Wilson et al., 2012), unless there is a cholinergic stimulation restoring phasic REM sleep (Hornung et al., 2007). Concerning sleep-dependent declarative memory consolidation processes, deficit has not been consistently found in the literature. An age-related decline in the consolidation of declarative memories has been associated with a decrease in early nocturnal SWS in middle-aged adults as compared to young adults (Backhaus et al., 2007). Differences in memory performance between young and middle-aged subjects disappeared when the amount of time spent in SWS was controlled for, stressing the importance of SWS in the consolidation process for declarative memories. Mander et al. (2013) reported that older adults do not benefit from post-learning sleep, unlike young adults who demonstrated better memory retention for learned word pairs after sleep. Davis et al. (2003) evidenced a more rapid forgetting after a 1-day delay in their oldest group (76–90 years) as compared to the youngest one (30–45 years). However, forgetting was not different in middle-aged (46–60 years) and older (61–75 years) groups as compared to the young one when all participants were matched for the acquisition rate during the learning phase. At variance with these studies, Aly and Moscovitch (2010) found a similar sleep-dependent benefit for the consolidation of declarative memory in young and older adults. This lack of age-related differences might be explained by the use of emotionally and personally relevant material, possibly leading to less forgetting than neutral material. Similarly, Wilson et al. (2012) reported a sleep-related declarative memory benefit for word pairs in young (20–34 years), middle-aged (35–50 years), and older (51–70 years) adults at 12 h post-learning, with an overall reduced forgetting rate after sleep. It must be noticed that most of these studies were using a word pairs learning procedure, but investigated age-related differences in declarative memory consolidation with a maximal 12-h delay after learning. Consequently, it remains unclear how a novel declarative material is consolidated in long-term memory, and possibly forgotten over longer time intervals ranging from several days to weeks. Moreover, it remains unclear at which stages age-related deficits occur in the formation of associative memories. To the best of our knowledge, long-term consolidation after days or weeks has never been studied in healthy aging. Therefore, this study investigated the time course of associative episodic memory formation in healthy young and older adults, in relation with subjective sleep quality. We hypothesized that although age-related deficits in memory consolidation may be concealed by normal performance patterns at learning and immediate recall, they would be expressed in an ALF phenomenon over a 7-day delay.

## MATERIALS AND METHODS

### PARTICIPANTS

Long-term declarative memory consolidation processes were investigated using a variant of the word pairs learning task in 16 young (range 18–30 years, *M* = 21.02, SD = 1.92; 2 men) and 16 older (range 65–75 years, *M* = 69.67, SD = 2.52; six men) healthy French-speaking adults. None of our participants took any medication that might affect sleep or memory and had a history of neurological, psychiatric, or sleep disorders. They all had normal or corrected vision. Health condition as self-reported on a Likert rating scale ranging from 0 (very bad) to 10 (excellent) did not significantly differ between groups [*t*(30) = 0.42; *p* = 0.68; young: 8.16 ± 0.85; older: 8.03 ± 0.85]. Depression scores on the shortened version of the Beck Depression Inventory (Beck et al., 1974; French translation by Collet and Cottraux, 1986) were also similar in both groups [*t*(30) = -0.55; *p* = 0.59; young: 2.19 ± 2.37; older: 2.69 ± 2.8], as well as sleep habits assessed using the Pittsburgh Sleep Quality Index (PSQI; adapted from Buysse et al., 1989; *t*(30) = -1.03; *p* = 0.3; young: 4.38 ± 2.09; older: 5.38 ± 3.28). All participants had neutral or intermediate chronotype, as measured by the Morningness–Eveningness Questionnaire (MEQ; Horne and Ostberg, 1976). Older participants had an intermediate morning-type (64.16 ± 6.56) whereas young adults had a neutral type [51.22 ± 10.52; *t*(27) = -4.18, *p *< 0.001]. Additionally, older participants were screened for dementia and scored within the normal range (score ≥ 26, 27.94 ± 1.3) at the Montreal Cognitive Assessment (MoCa; Nasreddine et al., 2005). This study was approved by the institutional ethics committee (Université Libre de Bruxelles).

### WORD PAIRS LEARNING TASK WITH ASSOCIATED PICTURES

**FIGURE 1:**
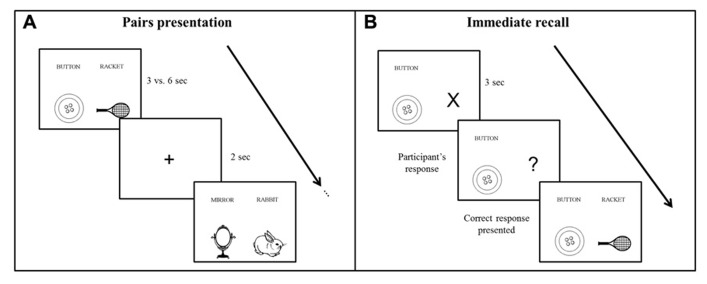
**Schematic representation of the word pairs learning procedure.** **(A)** During the encoding phase, participants viewed the word pairs by block of four pairs. Each pair was presented during 3 vs. 6 s for young and older participants, respectively. **(B)** During the immediate and delayed recall, the first word of the pair was given as a cue and participants responded with the corresponding word. Feedback was provided in the immediate recall phase only.

Participants learned a list of 28 semantically unrelated French word pairs, composed of concrete, high frequency (Desrochers and Bergeron, 2000; Bonin et al., 2003), and emotionally neutral bisyllabic nouns (Bonin et al., 2003; Syssau and Font, 2005). Each word pair was presented side by side on a computer screen, and each word was coupled with a corresponding schematic picture displayed below the word to facilitate associative imagery (see **Figure 1** for an example). Participants were explicitly instructed to use a mental imagery strategy to learn the association between the words of each pair, and older participants had more time to process the stimuli (see below), in order to compensate possibly less efficient strategic processes in aging (Dunlosky et al., 2005; Naveh-Benjamin et al., 2007). Performance was tested using a cued recall procedure immediately after the end of learning, 30 min later and after 7 days. A recognition test was also administered on day 7.

### SUBJECTIVE SLEEP QUALITY

The French version of the St Mary’s Hospital Sleep Questionnaire (Ellis et al., 1981) was administered to evaluate sleep during the night preceding both testing sessions. Sleep duration and latency measures were computed and compared with PSQI reports to test for the maintenance of sleep habits during the nights preceding testing sessions. Additionally, participants completed a daily sleep diary during the week separating learning from the retest 7 days later. Collected variables were self-reported sleep quantity including sleep duration (hours), latency (minutes), number of intra-sleep awakenings, duration of intra-sleep awakenings (minutes), naps duration (minutes), as well as subjective sleep quality measures (all scored on a Likert-scale from 1 to 5) including morning alertness, sleep depth, early awakening, ease to wake-up, ease to fall asleep, and dream quantity. A mean score over the week was calculated for each variable.

### PROCEDURE

The learning and testing sessions were administered at an exact interval of 7 days, at the same time of the day to avoid circadian confounding effects on performance (Schmidt et al., 2007). The first session started with the encoding phase, during which subjects learned the word pairs by blocks of 4, i.e., seven blocks of learning in total (see **Figure 1A**). Each word pair was presented during 3 s for the young and during 6 s for the older participants to control for age-related speed processing issues (Salthouse, 1996), which may lead to less efficient cognitive processing, including memory encoding (Davis et al., 2003; Craik and Rose, 2012). Indeed, associative learning deficits have been often reported in aging (Chalfonte and Johnson, 1996; Naveh-Benjamin et al., 2003; Lowndes et al., 2008), which suggests that older people need more time to bind words together. Each word pair was followed by the presentation of a central fixation cross for 2 s. After each learning block, there was an immediate cued recall of the four pairs. The first word of the pair was coupled with an “X” that changed into a “?” after 3 s, prompting the participant to verbally respond by saying the second corresponding word of the pair. Subject self-paced the time taken to recall each pair. Independently of the response provided by the participant, the correct word pair combination was displayed on the computer screen after each recall (**Figure 1B**). This procedure was repeated a second time, with randomized word pairs presentation.

An immediate cued recall phase followed encoding. At immediate recall, the first words of each of the 28 word pairs were presented in random order, and participants had to provide the corresponding word for each pair. A feedback with the correct word pair combination was provided following each response. Word pairs correctly recalled twice were considered learned, and no longer presented in further learning. The list of remaining pairs was learned again until either (a) the learning criterion of 100% of correct responses was reached at least twice or (b) the maximum of five presentations of the list of word pairs was achieved. After a 30 min break, during which subjects completed questionnaires, a delayed recall testing was administered. The first words of each of the 28 word pairs were presented in random order and participants had to provide the corresponding word for each pair. The whole list of 28 word pairs was presented only once and no feedback on the response was provided after the participant’s answer.

During the 1-week interval between the 30-min and delayed testing sessions, participants were required to keep regular sleep patterns and asked to complete a daily sleep diary, from learning to retest on day 7. The second testing session occurred on day 7 following learning and started with a delayed recall, followed by a recognition test. The delayed recall was administered using the same procedure as the 30-min recall.

The recognition test included 14 learned word pairs randomly selected from the total of 28 learned pairs, 14 randomly rearranged pairs from the remaining learned pairs, and 14 totally new pairs. Rearranged pairs were built by pairing the first word of one learned pair to the second word of another learned pair, so that both words were previously learned but were not bound together during the learning phase. All pairs were presented in a random order. For each pair, subjects were asked to tell whether they had previously learned this exact word pair. According to the dual-process theory (Yonelinas, 2002), recognition judgments rely on two distinct processes: recollection and familiarity. Recollection is a controlled process that relies on the conscious retrieval of contextual details associated with the learned episode, whereas familiarity is a more automatic process based on the “feeling of knowing” that an episode was previously encountered, without any contextual retrieval. In the framework of the dual-process theory, we predicted that familiarity-based recognition should be sufficient to correctly reject the new pairs, but should lead to errors in accepting rearranged pairs as learned because both words of the pair are familiar. Correct rejection of rearranged pairs requires the additional recollection of contextual details from the learning phase.

Finally, the Mill Hill vocabulary scale (Deltour, 1993) was administered to the participants to exclude the possibility that differences in declarative memory performance could be due to differences in verbal abilities.

### STATISTICS

In the word pairs learning task, between groups comparisons (young vs. older participants) were performed on the following variables: (a) at encoding, the immediate recall score was computed as the percentage of word pairs correctly recalled twice; (b) at 30-min and 7-day delayed testing, recall score was computed as the percentage of correctly recalled word pairs; and (c) long-term forgetting rate was calculated by subtracting the 30-min recall score from the 7-day recall score.

Performance in the recognition task was computed using hit rate and false alarms (FAs). Hit rate was calculated by dividing the number of correctly recognized pairs by the total number of presented learned pairs. FAs correspond to word pairs incorrectly recognized as learned. Two variables were computed to investigate the impact of FAs associated with rearranged pairs and new pairs on the hit rate score: (1) the number of correct recognition - (rearranged FA + new FA)/2) and (2) the number of correct recognition - new FA. The first FA score is a global associative-recognition score taking into account both the proportion of rearranged pairs and the proportion of new pairs erroneously endorsed as previously learned. The second FA score only considers the proportion of new pairs erroneously accepted as previously learned. The comparison between both scores aims at determining whether participants used both familiarity and recollection-based judgment to avoid the FA, or if they preferentially used familiarity due to a recollection deficit. Impaired recollection may result in significantly more FA in the global FA score than in the new FA score.

Differences between young and older groups were tested using independent Student’s *t*-tests. Recall performances and FA scores were tested using repeated measures analyses of variance (ANOVA). Mauchly’s sphericity test was used to verify the sphericity assumption in repeated measures designs, and Greenhouse–Geisser corrected *p*-values were used where necessary. Statistical significance was set at *p* = 0.05.

Finally, analyses of sleep diaries were conducted using *t*-tests for independent samples for quantitative sleep measures (e.g., sleep duration and latency). For sleep qualitative measures (e.g., ease to wake-up), scored on a Likert-scale (from 1 to 5), the non-parametric Mann–Whitney *U*-test was used (see **Table 2**). Since 12 between-group comparisons increase the potential for type 1 errors, statistical thresholds were corrected for multiple comparisons using the Bonferroni method (corrected *p*-value *p*^corr^ < 0.004).

## RESULTS

### WORD PAIRS LEARNING TASK WITH ASSOCIATED PICTURES

Mean percentages and standard deviations for performance in the Word pair learning task are reported in **Table 1** for each group.

**Table 1:** Word pairs learning task results [mean values (standard deviations)] for young and older adults.

	Young	Older
Number of trials to reach learning criteria	3 (1.51)	3.06 (1.65)
**Cued-recalls (%)**		
Immediate	98.22 (3.19)	93.97 (12.21)
30 min delay	96.43 (5.38)	93.3 (11.36)
7 days delay	82.81*** (15.93)	52.67 (17.91)
Long-term forgetting rate (%)	-13.62*** (3.69)	-42.42 (15.21)
**Recognition test (%)**		
Hit rate	99.11 (2.44)	94.2 (10.82)
Global FA score	93.53 (7.4)	88.62 (8.17)
New FA score	98.66 (2.88)	96.87 (4.49)

Learning criteria correspond to 100% of correct responses or five consecutive learning trials. FA, false alarms. Global FA score = [number of correct recognition - (rearranged FA + new FA)/2], New FA score = (number of correct recognition - new FA). Significant between-group differences: ***p < 0.001.

**Table 2:** Subjective sleep quality measures [mean values (standard deviations)] for young and older adults.

	Young	Older
**PSQI**		
Sleep duration (h)	8.02** (1.18)	6.92 (1.05)
Sleep latency (min)	23.59 (20.45)	23.34 (26.87)
**St Mary’s Hospital sleep questionnaire**		
Sleep duration (h) – day 1	8.08** (1.21)	7.13 (1.11)
Sleep latency (min) – day 1	29.69 (31.81)	22.44 (26.68)
Sleep duration (h) – day 7	7.9** (0.84)	7 (1.05)
Sleep latency (min) –day 7	25.5 (20.38)	22.25 (30.77)
**Sleep diaries**^a^		
Sleep duration (h)	7.98*** (0.7)	6.73 (0.71)
Sleep latency (min)	20.57 (14.33)	21.59 (18.84)
Number of intra-sleep awakenings	0.31*** (0.37)	1.27 (0.68)
Duration of intra-sleep awakenings (min)	3.94*** (7.6)	35.44 (30.32)
Naps duration (min)	1.2 (4.28)	10.91 (21.44)
Sleep quality^b^	3.86 (0.65)	3.7 (0.65)
Morning alertness^b^	3.43 (0.81)	3.87 (0.71)
Sleep depth^b^	3.91 (0.68)	3.53 (0.68)
Early awakening^b^	9.89 (0.67)	3.91 (0.7)
Ease to wake-up^b^	2.64 (0.62)	1.73*** (0.77)
Ease to fall asleep	2.16 (0.7)	2.12 (0.87)
Dreams quantity^b^	2.35 (0.91)	1.74 (1.03)

*Sleep duration and sleep latency were assessed with the St Mary’s Hospital sleep questionnaire on day 1 of testing and on day 7 of testing.*

a Averaged measures during the 7 days are presented in the table for sleep diaries.

b Ordinal variables on a Likert-scale ranging from 1 to 5. Significant between-group differences: ***p ≤ 0.001; **p < 0.01.

#### Learning and cued recall performance

**FIGURE 2:**
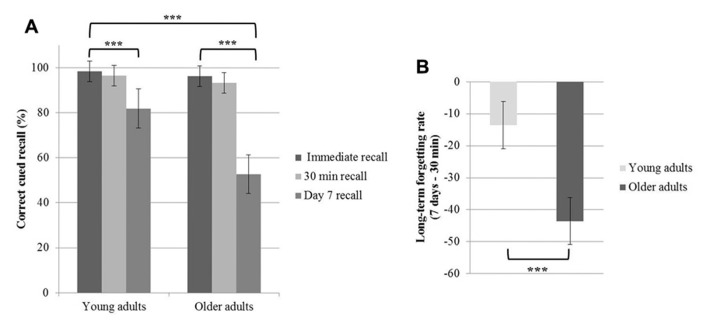
**Age-related changes in the word pairs learning task.** **(A)** Mean values of the percentage of correct cued recalls at immediate recall, 30 min recall and day 7 recall for the young and older participants. **(B)** Mean values of the long-term forgetting rate (difference between day 7 and 30-min recall performance). The error bars represent the 95% confidence intervals (***p < 0.001).****

A repeated measures ANOVA conducted on the percentage of correct responses during cued recall with the within-factor time of recall (immediate vs. 30 min vs. 7 days recall) and the between-factor group (young vs. older adults) disclosed main effects of time of recall [*F*(2,60) = 105. 5; *p *< 0.001] and group [*F*(1,30) = 12.92; *p *= 0.001], and a time of recall by group interaction effect [*F*(2,60) = 23.99; *p *< 0.001]. Tukey’s *post hoc* tests evidenced a significant decline in memory performance on day 7 both in young and older adults (*p* < 0.001). Additionally, *post hoc* tests evidenced a lower percentage of correct responses on day 7 in older than young adults (*p* < 0.001; **Figure 2A**). Immediate and 30 min delayed recall performances did not differ between groups (*p* > 0.9). The long-term forgetting rate, quantifying the decline from the 30-min recall to the 7-day recall phase, was significantly higher in older than young adults [*t*(30) = 5.63; *p* < 0.001, **Figure 2B**].

We tested the possibility that the forgetting rate observed on day 7 could be the consequence of differences in the quality of encoding at learning. Correlation analyses failed to reveal any association between the long-term forgetting rate and the immediate recall performance, either across all participants or within each group (all *r*^2^ < 0.038; *p* ≥ 0.3). Likewise, the number of trials needed to reach the 100% learning criteria was similar between groups [*t*(30) = -0.11; *p* > 0.9], as well as the percentage of correct responses at immediate recall (*p* > 0.9), suggesting equivalent encoding levels.

Older participants obtained better scores than the young ones at the Mill Hill vocabulary scale [*t*(30) = -2.05; *p* < 0.05; young: 32.31 ± 5.63; older: 36.5 ± 5.93], in line with findings evidencing a small improvement across the life span in verbal ability (Park et al., 2002). This result discards the possibility that a weaker verbal ability could explain the weaker delayed memory performance in the older group.

Our groups were not perfectly matched for sex as two males only were included in the young group, but six males in the older group. Nonetheless, correlation analyses failed to disclose any relationships between sex differences and long-term forgetting rate or long-term delayed recall across all participants (all *r*^2^ < 0.08; *p* > 0.11) or within each age group (all *r*^2^ < 0.04; *p* > 0.4), suggesting that sex differences did not impact long-term memory performance.

#### Recognition test

There was a trend for a lower hit rate in older than young participants [*t*(30) = 1.77; *p* = 0.086], indicating less accurate recognition of the learned pairs. A repeated measures ANOVA conducted on the percentage of correct recognition with the within-factor FA type (global vs. new FA) and the between-factor group (young vs. older adults) disclosed a main effect of FA type [*F*(1,30) = 33.96; *p *< 0.001] with more FA in the global FA score than in the new FA score, a trend for significance for the group effect [*F*(1,30) = 3.33; *p *= 0.078] with more FA during the recognition test for the elderly, but no FA type by group interaction [*F*(1,30) = 1.85; *p *= 0.18].

### SUBJECTIVE SLEEP QUALITY

Sleep duration and sleep latency were estimated using (a) the PSQI to evaluate participants’ sleep habits during the month preceding the testing, and (b) the St Mary’s Hospital sleep questionnaire to verify the maintenance of sleep habits during the nights preceding both testing sessions (**Table 2**). A repeated measures ANOVA performed on sleep duration with sleep duration measures (usual sleep duration vs. sleep duration before day 1 vs. sleep duration before day 7) as within-subjects factor and the group as between-subjects factor yielded a main group effect [*F*(1,30) = 9.94; *p *< 0.01] with shorter sleep duration in older than young participants, but no effect of sleep duration measures [*F*(1,30) = 0.39; *p *= 0.7], nor any interaction [*F*(1,30) = 0.15; *p *= 0.9], suggesting that the participants’ sleep habits were similar during the experiment and over the preceding month. Regarding sleep latency, repeated measures ANOVA performed on sleep latency with sleep latency measures (usual sleep latency vs. sleep latency before day 1 vs. sleep latency before day 7) as within-subjects factor and group as the between-subjects factor did not reveal any significant effects (*p*s > 0.6).

It is known that between groups difference in circadian time of testing or accumulated sleep pressure might exert an influence on memory performance (Schmidt et al., 2007). However, correlation analyses between circadian preferences (neutral type for the young vs. intermediate morning-type for the older adults) and delayed recall performance on day 7 or long-term forgetting rate in young or older participants were all non-significant (all *r*^2^ < 0.048; *p* ≥ 0.41). In addition, the mean elapsed time between wake-up time and time of testing was not significantly different between groups, neither for the first [*t*(30) = 0.2, *p* = 0.84], nor for the second session of testing [*t*(30) = 1.1, *p* = 0.28].

**FIGURE 3:**
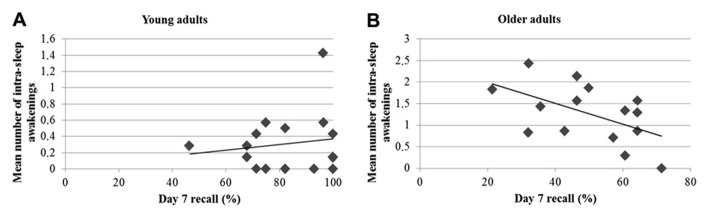
**Pearson’s correlations between the day 7 recall performance and the number of intra-sleep awakenings in young (A)** and older participants **(B)**.

Finally, analyses conducted on daily sleep diaries revealed that older adults experienced on average a shorter sleep duration [*t*(30) = 5.03; *p* < 0.001], more sleep awakenings [*t*(29) = -4.95; *p* < 0.001], and longer sleep awakenings [*t*(30) = -4.03; *p* < 0.001] than young adults. However, older participants felt it was easier to wakeup than young participants (*U* = 42.5; *p* = 0.001). Separate correlational analyses were computed between the 7-day recall performance and mean sleep duration, mean number, and duration of sleep awakenings during the 7 days. Correlations between long-term recall and sleep duration and duration of sleep awakenings were weak and non-significant (all *r*^2^ < 0.15; *p* > 0.15). Only, the number of intra-sleep awakenings was negatively correlated with delayed recall scores on day 7 in the older group (*r* = -0.53; *p* = 0.04), but not in the young group (*r* = 0.15; *p* = 0.59). As shown in **Figure 3**, there was a trend for a significant difference between young and older participants in the association slopes between the number of intra-sleep awakenings and recall performance at 7 days (*p* = 0.075). However, the significant correlation between the mean number of intra-sleep awakenings and the long-term recall performance in the older group is not present anymore when corrected for multiple comparisons (corrected *p*-value *p*^corr^ < 0.017). Furthermore, the long-term forgetting rate was not related to any of the selected sleep variables (all *r*^2^ < 0.11; *p* > 0.22).

## DISCUSSION

This study investigated the formation and consolidation of associative episodic memories in healthy young and older adults in relation with subjective sleep quality. Our results evidence an ALF at memory retrieval in aging, 7 days after learning, which may be partially related to increased sleep fragmentation. Older and young participants did not differ in performance at encoding in the learning task, suggesting a genuine consolidation deficit. ALF in aging is consistent with previous reports suggesting an age-related decline in the consolidation of declarative memory after 10 h including a period of sleep (Mander et al., 2013), after a 1-day interval (Davis et al., 2003) or after an early night of sleep rich in SWS (Backhaus et al., 2007). At variance, Aly and Moscovitch (2010) and Wilson et al. (2012) did not evidence differences in sleep-dependent memory consolidation between young and older adults. Here, we used an unrelated word pairs learning task like Wilson et al. (2012). However, these authors tested memory retrieval after a 12-h delay only. This methodological difference and the lack of consistency between studies when delayed retest is up to 12 h suggest that a longer retention interval might be more sensitive to evidence age-related differences in memory consolidation. In this respect, forgetting was not increased after a 1-day delay in the 61–75 years group compared to the 30–45 years group in the Davis et al. (2003) study, whereas ALF was evidenced in the oldest (76–90 years) group. Since we found ALF to be present in the 65- to 75-years-old age range, we surmise that a longer time period between learning and delayed test phases might enhance the detection of early age-related differences in memory consolidation. Finally, our findings are in agreement with the idea that memory consolidation processes do not exclusively depend on the quality of sleep during the first night after learning, and that changes may continue to take place during the days following learning (Dudai, 2004).

Analyses on self-reported sleep measures disclosed quantitative and qualitative differences between older and young participants. Indeed, older subjects reported shorter sleep duration as well as an increased frequency of intra-sleep awakenings that lasted for longer than in young subjects, as previously reported (Ohayon et al., 2004). Older subjects also reported less difficulty to wake-up in the morning, which can be partially associated with their intermediate morning-type as measured by the MEQ (Horne and Ostberg, 1976). Furthermore, correlational analyses in the older group evidenced that the delayed recall performance after 7 days is negatively related to the number of intra-sleep awakenings during the intermediary week. There was a trend for a difference in this association between young and older groups suggesting that sleep fragmentation partly contributes to decreased levels in recall performance after retention for a week. However, other studies suggest that the intra-sleep awakenings *per se* do not affect memory. Indeed, Ficca et al. (2000) fragmented sleep in young participants and evidenced that delayed overnight memory performance was only altered when sleep fragmentation was associated with a disruption in sleep cycles. This result suggests that memory consolidation processes would be impaired by disorganization in sleep cycles induced by sleep fragmentation, but not by sleep fragmentation *per se*. Sleep fragmentation in older adults might also be related to an increased amount of time spent in light sleep, and a decreased time spent in SWS (Ohayon et al., 2004), that plays an important role in stabilizing or even enhancing retention levels in episodic memory (Plihal and Born, 1997; Tucker and Fishbein, 2008). Previous studies have evidenced an age-related decline in declarative memory consolidation associated with reduced SWS (Backhaus et al., 2007; Mander et al., 2013). Furthermore, when the amount of SWS were equated in middle-aged and young participants, retention scores after sleep were similar in both age groups, indicating that SWS plays a prominent role in declarative memory consolidation independently of age (Backhaus et al., 2007), and that SWS decline due to sleep fragmentation in aging might exert a deleterious impact on sleep-dependent memory consolidation processes.

It has been proposed that sleep preferentially supports the consolidation of memories that are of future relevance. Participants who, after learning, were informed about a later test of their memory performance showed enhanced sleep-dependent memory consolidation as compared to participants who did not expect a subsequent test (Wilhelm et al., 2011). In the present study, we informed our participants that their memory performance would be retested 1 week later. We also asked them to refrain from writing or rehearsing the word pairs during the week. However, it can be argued that learning unrelated word pairs was not relevant for our participants’ everyday life, and therefore that these pairs might not have been reinforced over the course of days following learning. Furthermore, declarative memory has been shown to be more vulnerable to forgetting (Diekelmann et al., 2009; Stickgold, 2013), which may explain why the information was partially forgotten in both groups.

Another factor modulating the benefits of sleep on the consolidation of word pairs learning is the strength of initial encoding. For instance, high performers at learning exhibited a beneficial effect of a daytime nap on memory performance whereas low performers did not perform better following a nap than following a period of wake (Tucker and Fishbein, 2008). Others have reported higher post-learning sleep gains for weak than strong memory traces (Drosopoulos et al., 2007). In the present study, the immediate recall performance and the number of trials to reach the learning criteria were comparable, suggesting similar amounts of learned information and similar depth of encoding in the young and older groups. Moreover, both groups reached comparable performance levels at immediate and short-delay (30 min) testing sessions. Considering that encoding levels were controlled for, we propose that our finding of ALF in aging cannot be explained by differences in individual encoding capacities. However, we cannot exclude that the neural processes involved during encoding might be different in young and older participants, therefore impacting subsequent consolidation processes. For instance, items associated with higher hippocampal activity at encoding have been found to be more strongly consolidated following a night of sleep (Rauchs et al., 2011). Hippocampal activation at encoding might reflect the tagging of relevant or irrelevant memories and drive the neural reactivation during the subsequent sleep, by promoting either remembering or forgetting (Rauchs et al., 2011; Stickgold and Walker, 2013). Although hippocampal activity at encoding was similarly related to recognition performance in young and older adults, functional connectivity between the hippocampus and frontal regions has been shown to shift from ventral to dorsal frontal areas with aging (Grady et al., 2003). The authors have suggested that interactions between these brain regions at encoding might facilitate subsequent recognition performance in the different age groups, but that functional network differences may imply the additional involvement of executive functions in older adults in order to maintain and reinforce memory performance. Considering the hypothesis that neural tagging of memory at encoding promotes memory replay during sleep, it can be proposed that tagging processes, and the subsequent memory consolidation processes will both be differentially handled in aging due to age-related changes in hippocampal activation and functional connectivity at encoding. Future research measuring functional activity at both encoding and long-term retrieval in aging is needed to verify this tentative explanation.

In the recognition task, our older group performed better than in the recall task, and almost equally well than the young subjects. Together with previous findings, this outcome strongly suggests that the observed decline in long-term recall performance is not due to an age-related deterioration of encoding mechanisms in healthy aging. Difficulties might rather come from a strategic nature (Lowndes et al., 2008). For instance, the elderly may fail, or merely be worse at implementing reproduction strategies when trying to recall newer and less associated information (Dunlosky et al., 2005; Naveh-Benjamin et al., 2007). However, we found that young and older participants were similarly able to recall word pairs 30 min after learning. In this respect, providing specific instructions to use appropriate strategies to bind word pairs together at encoding may have limited associative deficits in aging in the short-term, as previously reported (Naveh-Benjamin et al., 2007). In addition, both groups tended to exhibit more FAs for the rearranged than for the new pairs. The use of familiarity-based recognition strategies following recollection deficits may contribute to the increased number of errors for the rearranged pairs. In the present study, the interaction between the type of FAs and group was not significant, although older participants tended to globally produce more FAs than the young ones. As older adults have been assumed to rely more on familiarity than recollection processes (see Yonelinas, 2002, for a review), more FAs might have been expected for the rearranged pairs. However, increased difficulties to recollect information in the long-term, as evidenced by a decreased recall performance in older participants, was not observable in the recognition test. This effect might be due to near ceiling effects in recognition performance due to our strengthened, imagery-based encoding strategy, and/or to the administration of the cued-recall just before the recognition test.

Akin to the present study, memory consolidation in aging was assessed for implicit learning across three different retention intervals in young and older participants: 12 h, 24 h, and 7 days (Nemeth and Janacsek, 2010). Results evidenced an offline improvement in general motor skill performance in the older group only after 12 h, even though this improvement was weaker than in young adults. Offline enhancement was also found in young adults following the 24-h and the 1-week delays, but not in the older population. Additionally, sequence-specific learning remained stable in the young group, but decreased similarly for both the 24-h and the 1-week delays in the older group. These findings suggest the existence of age-related differences in the time course of the consolidation of implicit sequence learning. Although a prior study from the same group suggested that sleep does not play a critical role in the consolidation of implicit sequence learning in both young and old adults tested after a 12-h interval (Nemeth et al., 2010; but see Urbain et al., 2013), it is difficult to draw firm conclusions regarding the involvement of age-related changes in sleep measures on long-term implicit memory consolidation processes, especially after a 7-day interval as these measures were not investigated in the Nemeth and Janacsek (2010) study. Using an explicit procedural memory task, Peters et al. (2008) evidenced memory enhancement both in young and old adults when tested 7 days after the initial learning, even though the enhancement was more pronounced for the young group. Other studies focusing on procedural learning have assumed that memory difficulties in the elderly are not present during the initial encoding phase, but actually only appear later on when sleep-dependent memory consolidation processes take place (Spencer et al., 2007; Brown et al., 2009; Wilson et al., 2012). However, these studies did not investigate changes in sleep measures in association with a decline in procedural memory consolidation, leaving the unanswered question of the contribution of sleep features to ALF (or consolidation) over several days. It has been suggested that procedural memory consolidation preferentially benefits from REM sleep (Plihal and Born, 1997) or from increased density of NREM sleep spindles (Fogel and Smith, 2006). Accordingly, studies in aging have shown that artificially increasing cholinergic levels during REM sleep promotes an overnight improvement of procedural memory consolidation (Hornung et al., 2007), and that the density of sleep spindles following procedural learning does not increase in older adults as observed in young adults (Peters et al., 2008). Future research needs to investigate the parallels and discrepancies between age-related long-term forgetting and consolidation processes for declarative and non-declarative memories.

Finally, it is fair to mention potential limitations in this study. Sleep measures obtained using daily sleep diaries must be interpreted with caution as they represent subjective measurements that may be biased by different response strategies. Polysomnographic recordings would allow objective measurements of sleep variables associated with changes in sleep architecture, and further probing the assumption that increased sleep fragmentation and ALF in aging are related to increased time spent in light sleep, and decreased time spent in SWS. Despite these limitations, the results of the present study may have some clinical implications. First, our data support the idea that an adapted treatment improving sleep quality or sleep architecture may help to reduce ALF in aging, in line with prior demonstrations (Hornung et al., 2007) and proposals (Mander et al., 2013). Second, our results suggest that sleep-dependent memory consolidation is not achieved during the first post-learning night. Consolidation processes rather continue to reorganize the novel memory traces across several days following learning. This should encourage the investigation of memory consolidation processes after delays above 12 h. Third, our finding of an ALF in a healthy older population stresses the sensitivity of long-term forgetting measures to evidence consolidation deficits in normal aging. Our results therefore encourage the use of long-term forgetting measures in clinical populations, particularly in the case of patients reporting subjective memory complaints but with normal performance at standard neuropsychological testing. Indeed, a standard neuropsychological assessment usually tests the retention of the so-called long-term memory (as opposed to short-term and working memory processes) after a period of 30 min to 1 h. This limited retention span might be simply too short to evidence a memory decline, and further a consolidation deficit in a pre-clinical population with memory complaints (Manes et al., 2008) eventually leading to an underestimation in diagnoses of memory disorders. Measuring ALF might therefore be of clinical importance in the everyday neuropsychological testing of long-term memory, as well as the investigation of sleep quality and quantity in patients. Fourth, prior reports suggest that age-related deficit in memory are already detectable in middle-aged adults (Davis et al., 2003; Backhaus et al., 2007). Given the importance to evidence early memory deficits for clinical diagnosis, it seems therefore essential to determine whether ALF measures are similarly sensitive in the middle-age range.

To sum up, the present study evidenced an exaggerated deficit in the recall of associative episodic memories over a 7-day interval in healthy older subjects, as compared to a young population, despite similar encoding levels and recall performance when tested half an hour later. Furthermore, our results suggest that age-related ALF may be at least partially associated with increased sleep fragmentation, as suggested by a negative correlation between delayed recall performance and increased frequency of intra-sleep awakenings in older participants.

## Conflict of Interest Statement

The authors declare that the research was conducted in the absence of any commercial or financial relationships that could be construed as a potential conflict of interest.

## Author Contributions

Alison Mary designed the study, conducted the experiment, analyzed the data and wrote the manuscript. Svenia Schreiner aided in study design, conducted the experiment and aided in data analyses and manuscript writing. Philippe Peigneux designed the study, aided in data analyses and wrote the manuscript.
